# Supplementary material for: Ultrastructural heterogeneity of layer 4 excitatory synaptic boutons in the adult human temporal lobe neocortex
Source: eLife. 2019 Nov 20;8:e48373. doi: 10.7554/eLife.48373 (PMC6919978; doi:10.7554/eLife.48373)
Supplement: Source data 1. [file elife-48373-data1.docx]

| **Patients** | **Dis**  **1** | **Dis**  **2** | **Dis**  **3** | **Dis**  **4** | **Dis**  **5** | **Dis**  **6** | **Dis**  **7** | **Dis**  **8** | **Dis**  **9** | **Dis**  **10** | **Dis**  **11** | **Dis**  **12** | **Dis**  **13** | **Dis**  **14** | **Dis**  **15** | **Dis**  **16** | **Dis**  **17** | **Dis**  **18** | **Dis**  **19** | **Dis**  **20** | **Syn**  **Dens** |
| --- | --- | --- | --- | --- | --- | --- | --- | --- | --- | --- | --- | --- | --- | --- | --- | --- | --- | --- | --- | --- | --- |
| **Hu_1** | 0 | 0 | Ex/sp | 0 | Ex  /  Sp | In  /Sp | 0 | 3Ex  /Sh | 0 | 0 | 0 | 0 | 2Ex  /Sp | 0 | Ex  /Sp | Ex  /Sp | 0 | Ex  /Sp | 2Ex/  Sp  Ex/  Sh | Ex  /Sh | 0,0041  = 0,41/  100µm^3^  **Total**  4.1*10^6^/mm^3^ |
| **Hu_2** | 0 | 2  Ex/  Sp | Ex/  Sh | 0 | 0 | 0 | Ex/  Sp | 0 | Ex/  Sh | 0 | 0 | 0 | 0 | 0 | 0 | 0 | 0 | 0 | 0 | 0 | 0,0013  =  0,13/  100 µm^3^  **Total**  1.3*10^6^/mm^3^ |
| **Hu_3** | 4  Ex/  Sp  Ex/  Sh  2  In/  1  Sh | 5  Ex/  Sp  2  Ex/1  Sh | 0 | 0 | 0 | Ex/  Sp | 3  Ex  /  Sp | 0 | 0 | Ex/  Sp | 2  Ex/  Sp | Ex/  Sp | Ex/  Sh | 0 | 0 | 0 | 0 | 0 | Ex/  Sp | Ex/  Sp | 0,006=  0,6/  100  µm^3^  **Total**  6*10^6^/mm^3^ |
| **Hu_4** | Ex/  Sp | 0 | 0 | 0 | 0 | 0 | 0 | Ex/  Sp | Ex/  Sp | 0 | 0 | 0 | 0 | 0 | 0 | 0 | 0 | 0 | 0 | 0 | 0,0008=  0,08/  100 µm^3^  **Total**  0.8*10^6^/mm^3^ |
| **Hu_5** | 0 | 0 | Ex/  Sp | 0 | 0 | 0 | 0 | 0 | 0 | 0 | 0 | 0 | 0 | 0 | 0 | 0 | 0 | 0 | Ex/  Sp | 0 | 0,0005=  0,05/  100 µm^3^  **Total**  0.5*10^6^/mm^3^ |
| **Hu_6** | 0 | Ex/  Sp | Ex/  Sh | Ex  /  Sh  Ex  /  Sp | 0 | 0 | 0 | 0 | 0 | 0 | 0 | 0 | 0 | 0 | 0 | Ex  /  Sp | 0 | Ex  /  Sp | 0 | 0 | 0,0015  =  0,15/  100 µm^3^  **Total**  1.5*10^6^/mm^3^ |

Dis: Dissector

Ex: Excitatory

In: Inhibitory

Sp: Spine

Sh: Shaft
