# Supplementary material for: Ultrastructural heterogeneity of layer 4 excitatory synaptic boutons in the adult human temporal lobe neocortex
Source: eLife. 2019 Nov 20;8:e48373. doi: 10.7554/eLife.48373 (PMC6919978; doi:10.7554/eLife.48373)
Supplement: Transparent reporting form [file elife-48373-transrepform.pdf]

eLIFE
1st Floor
24 Hills Road
Cambridge CB2 1JP, UK
P 01223 855340
W elifesciences.org
T @elife

# *eLife's* transparent reporting form

We encourage authors to provide detailed information *within their submission* to facilitate the interpretation and replication of experiments. Authors can upload supporting documentation to indicate the use of appropriate reporting guidelines for health-related research (see EQUATOR Network), life science research (see the BioSharing Information Resource), or the ARRIVE guidelines for reporting work involving animal research. Where applicable, authors should refer to any relevant reporting standards documents in this form.

If you have any questions, please consult our Journal Policies and/or contact us: editorial@elifesciences.org.

**Sample-size estimation**

- You should state whether an appropriate sample size was computed when the study was being designed
- You should state the statistical method of sample size computation and any required assumptions
- If no explicit power analysis was used, you should describe how you decided what sample (replicate) size (number) to use

Please outline where this information can be found within the submission (e.g., sections or figure legends), or explain why this information does not apply to your submission:

- Sample size: In this study we used neocortical access tissue from the temporal lobe taken from three adult female and three male patients that underwent epilepsy surgery. Additional and more detailed information could be found under section Material and Methods/Human neocortical tissue processing for EM and in Table 3 pg. 31; and Golgi-Cox impregnation of biopsy material pg. 34. One additional female patient was used for Focused ion beam scanning electron microscopy. See Material and Methods/Focused ion beam scanning electron microscopy pg. 36.
- Statistics: The non-parametric Kruskal-Wallis H-test analysis was computed, as some of the analyzed parameters were not normally distributed as indicated by the skewness. For details, see Material and Methods/Statistical analysis pg. 39.
- Power analysis: For the present study, it was not applicable because of the limited availability of well-preserved neocortical tissue samples.

**Replicates**

- You should report how often each experiment was performed
- You should include a definition of biological versus technical replication
- The data obtained should be provided and sufficient information should be provided to indicate the number of independent biological and/or technical replicates
- If you encountered any outliers, you should describe how these were handled
- Criteria for exclusion/inclusion of data should be clearly stated
- High-throughput sequence data should be uploaded before submission, with a private link for reviewers provided (these are available from both GEO and ArrayExpress)

eLife Sciences Publications, Ltd is a limited liability non-profit non-stock corporation incorporated in the State of Delaware, USA, with company number 5030732, and is registered in the UK with company number FC030576 and branch number BR015634 at the address 1st Floor, 24 Hills Road, Cambridge CB2 1JP | August 2014

Please outline where this information can be found within the submission (e.g., sections or figure legends), or explain why this information doesn't apply to your submission:

- For technical and biological replication, neocortical tissue samples from six patients were processed for transmission electron microscopy, Golgi-Cox impregnation, stereological estimation of synaptic contacts density and tomography. See Material and Methods/ Human neocortical tissue processing for EM pg. 30; Golgi-Cox impregnation of biopsy material pg. 34; Stereological estimation of the density of synaptic contacts pg. 35; EM tomography of L4 SBs in the TLN pg. 38. See also Material and Methods/ 3D-volume reconstructions and quantitative analysis of L4 SBs pg. 36; cluster analysis (CA) of excitatory SBs in L4 of the TLN pg. 39. One tissue sample from an additional female patient was processed for focused ion beam scanning electron microscopy. See Material and Methods/Focused ion beam scanning electron microscopy pg. 36.
- In this study, all data were used in the analysis, including outliers. See correlation graphs (Figs. 5-6, pgs. 17-18) and boxplots (Fig. 11, pg. 33).
- High-throughput sequence data were not applicable in our submission; as we do not deal with genomic sequences.

**Statistical reporting**

- Statistical analysis methods should be described and justified
- Raw data should be presented in figures whenever informative to do so (typically when N per group is less than 10)
- For each experiment, you should identify the statistical tests used, exact values of N, definitions of center, methods of multiple test correction, and dispersion and precision measures (e.g., mean, median, SD, SEM, confidence intervals; and, for the major substantive results, a measure of effect size (e.g., Pearson's r, Cohen's d)
- Report exact p-values wherever possible alongside the summary statistics and 95% confidence intervals. These should be reported for all key questions and not only when the p-value is less than 0.05.

Please outline where this information can be found within the submission (e.g., sections or figure legends), or explain why this information doesn't apply to your submission:

- For statistical analysis, see Material and Methods/Statistical analysis pg. 39.
- In the present study, N (representing the number of synaptic boutons analyzed) per group is larger than 10.
- For each experiment, H-test was applied and all statistical values were given in Table 2 pg. 12; Fig. 11 pg. 33 and throughout the text where appropriate.
- Exact p-values were given where appropriate.

(For large datasets, or papers with a very large number of statistical tests, you may upload a single table file with tests, Ns, etc., with reference to sections in the manuscript.)

**Group allocation**

- Indicate how samples were allocated into experimental groups (in the case of clinical studies, please specify allocation to treatment method); if randomization was used, please also state if restricted randomization was applied
- Indicate if masking was used during group allocation, data collection and/or data analysis

Please outline where this information can be found within the submission (e.g., sections or figure legends), or explain why this information doesn't apply to your submission:

- Only for the density of synaptic contacts measurements, samples were allocated into two experimental groups (Males and Females).
- Data collection and analysis were unbiased.

**Additional data files ("source data")**

- We encourage you to upload relevant additional data files, such as numerical data that are represented as a graph in a figure, or as a summary table
- Where provided, these should be in the most useful format, and they can be uploaded as "Source data" files linked to a main figure or table
- Include model definition files including the full list of parameters used
- Include code used for data analysis (e.g., R, MatLab)
- Avoid stating that data files are "available upon request"

Please indicate the figures or tables for which source data files have been provided:

- We will upload the files as requested.
- Figures and tables for which source data files have been provided: Table 2, Figs. 4, 5, 6, 8 and 11.
